# Supplementary material for: The gut microbiome in atherosclerotic cardiovascular disease
Source: Nat Commun. 2017 Oct 10;8:845. doi: 10.1038/s41467-017-00900-1 (PMC5635030; doi:10.1038/s41467-017-00900-1)
Supplement: Supplementary file 1 — Supplementary Information [file 41467_2017_900_MOESM1_ESM.pdf]

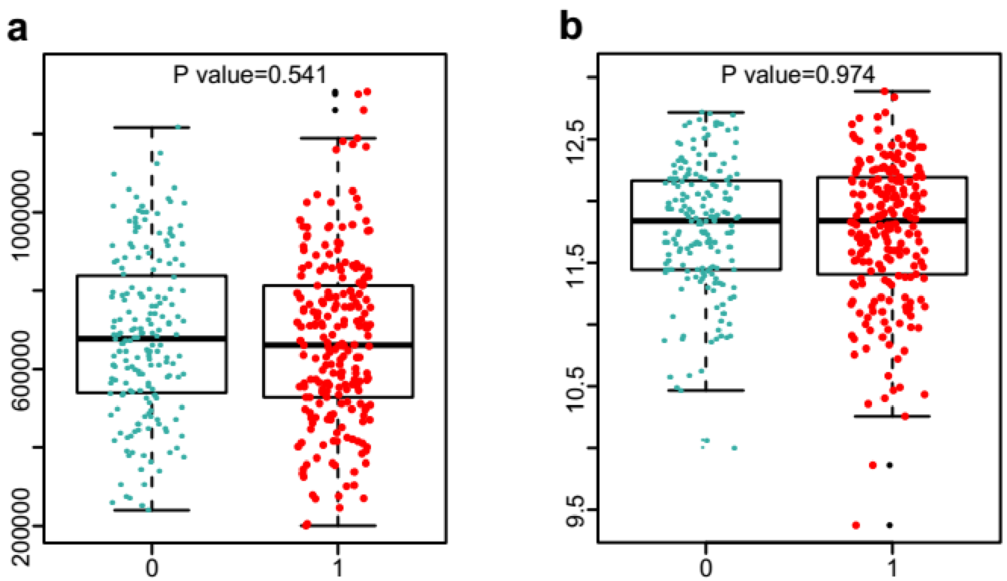

Supplementary Figure 1. Diversity and richness of the ACVD gut microbiome.
(a) Gene richness of the ACVD patients (n= 218, red) compared to the control groups (n=187, cyan). p-values from Wilcoxon rank-sum test are shown. **(b)** α-diversity (Shannon index) of the ACVD patients (n= 218, red) and control groups (n=187, cyan). Wilcoxon rank-sum test.

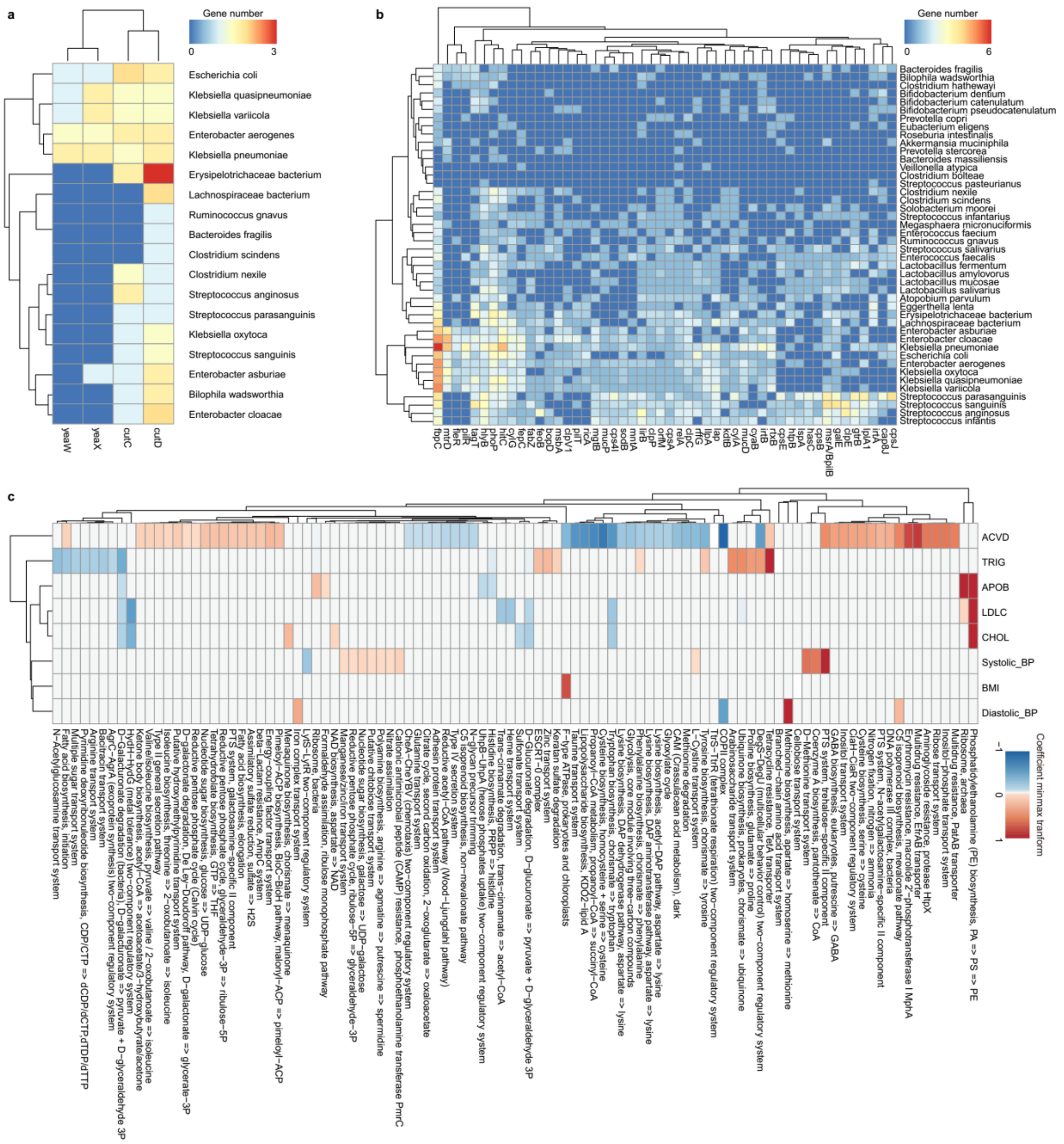

Supplementary Figure 2. Functions encoded by ACVD-associated species.
(a) ACVD microbiome containing TMA-lyases. PanPhlAn was used to determine the presence or absence of genes in each sample that belong to bacterial species identified as differentially enriched MLGs (**Fig. 2**, **Supplementary Data 3**). The heatmap was colored according to the number of genes that showed a significant difference in occurrence between ACVD cases (n=218) and controls (n=187) (Fisher test, q-value < 0.05). **(b)** Virulence factors in the ACVD microbiome according to the VFDB database. PanPhlAn as in panel **a.** Virulence factors that were found in less than 7 species are not shown. Colored according to the square root of the number of genes that showed a significant difference in occurrence between ACVD cases (n=218) and controls (n=187) (Fisher test, q-value <0.05). **(c)** KO modules characteristic of ACVD or related clinical indices. All modules were regressed against the index using group LASSO. The heatmap was colored according to coefficients from bootstrapping cross-validation group LASSO, after min-max transformation.

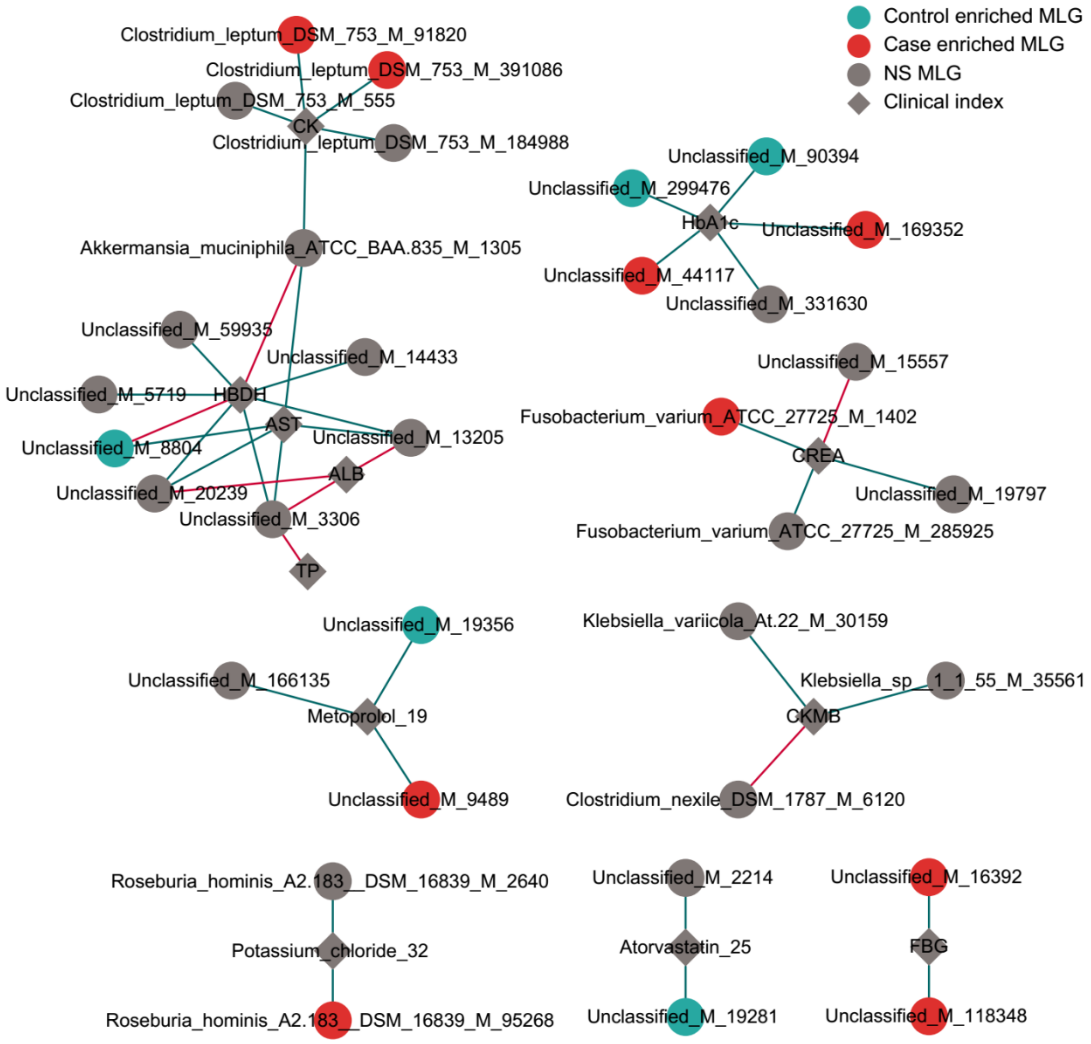

Supplementary Figure 3. MLGs influenced by drugs or clinical indices.
Associations in ACVD patients identified by MaAsLin. Green edges, positive associations; red edges, negative associations. Red circles, ACVD-enriched; cyan circles, control-enriched; grey circles, no significant difference. q-value < 0.05 between 187 healthy controls and 218 ACVD cases, FDR controlled Wilcoxon rank-sum test.

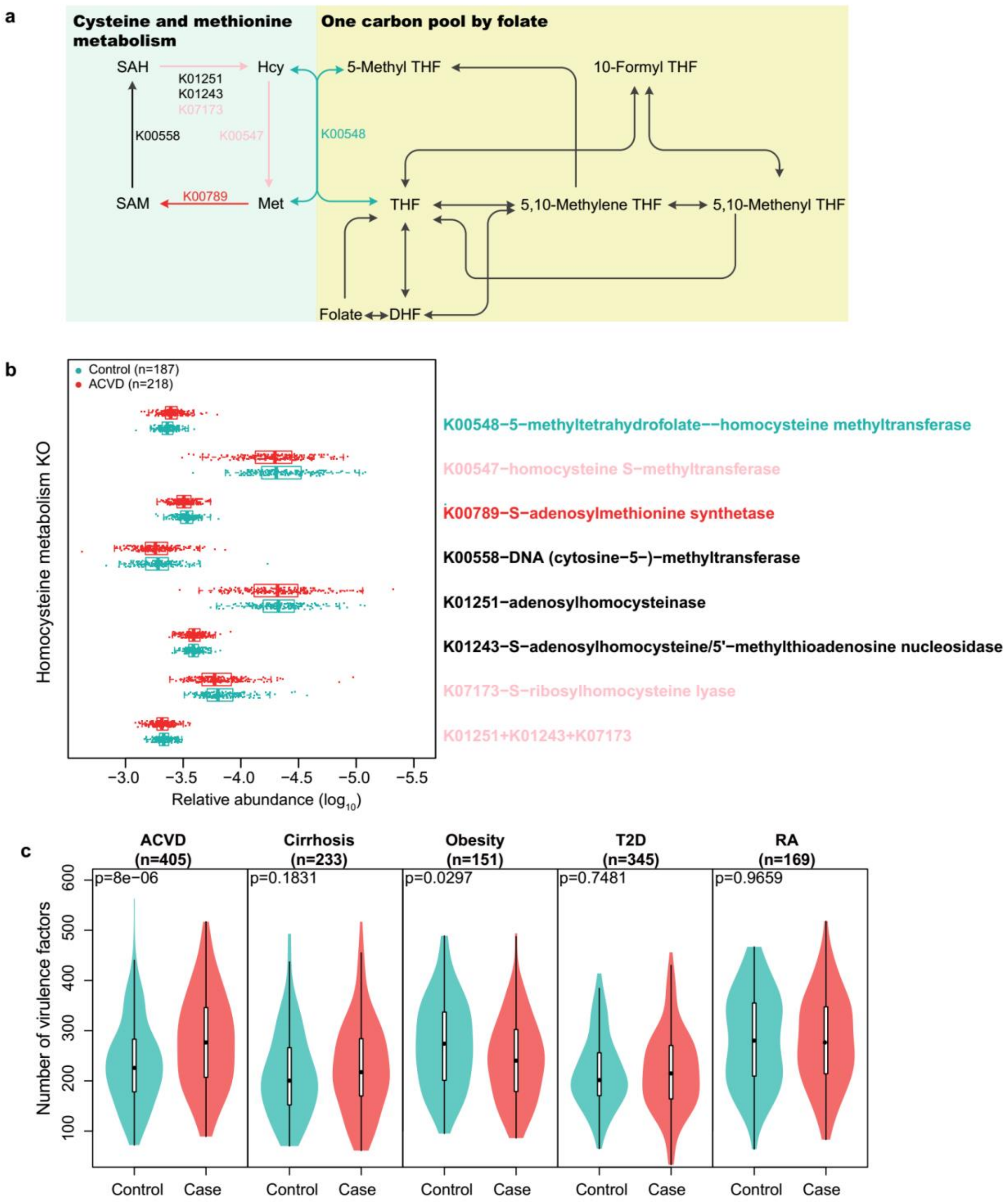

Supplementary Figure 4. Homocysteine metabolism and virulence factors.
(a) Pathways for homocysteine and folate metabolism. **(b)** Relative abundances of the KOs involved in homocysteine metabolism. The KOs were colored according to significant differences between the ACVD samples (n= 218) and control samples (n=187), i.e. red or cyan, p-value < 0.01; light red or green, p-value < 0.05; black, p-value ≥ 0.05, Wilcoxon rank-sum test. **(c)** Number of virulence factors in the cohorts. Counted in each sample according to VFDB, p-values from Wilcoxon rank-sum tests. Among these, RA was the only cohort from northern China, and obesity was the only young adult cohort.

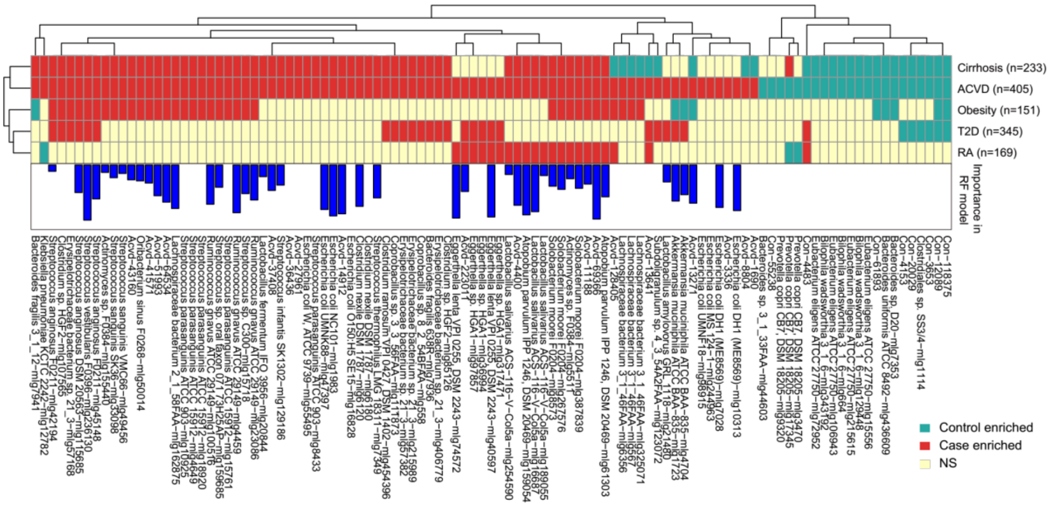

Supplementary Figure 5. Presence of ACVD marker MLGs in other disease cohorts. Red, case-enriched; cyan, control-enriched; yellow, no significant difference, Wilcoxon rank-sum test, q-value < 0.05.

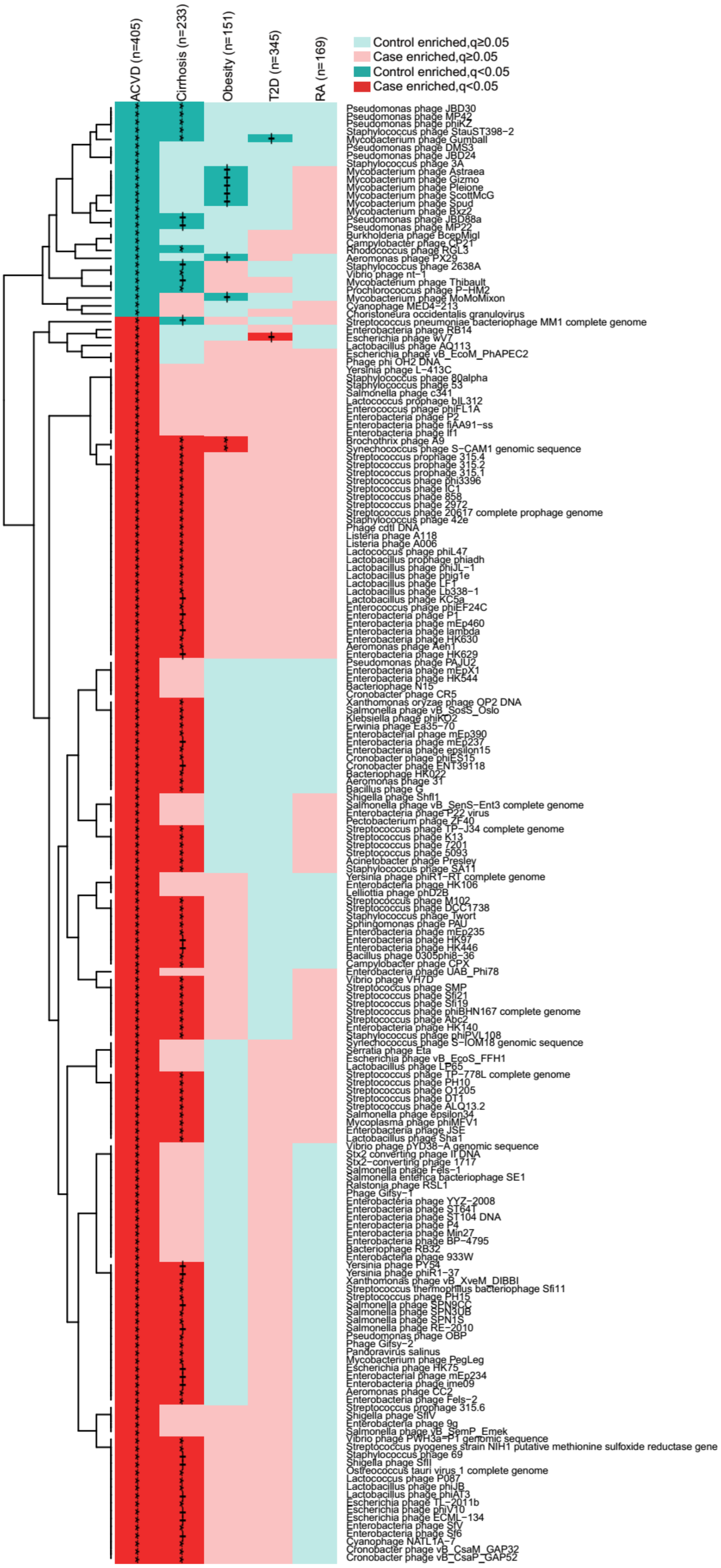

ACVD (n=405)
Cirrhosis (n=233)
Obesity (n=151)
T2D (n=345)
RA (n=169)
Control enriched,q≥0.05
Case enriched,q≥0.05
Control enriched,q<0.05
Case enriched,q<0.05
Pseudomonas phage JBD30
Pseudomonas phage MP42
Pseudomonas phage phiKZ
Staphylococcus phage StauST398−2
Mycobacterium phage Gumball
Pseudomonas phage DMS3
Pseudomonas phage JBD24
Staphylococcus phage 3A
Mycobacterium phage Astraea
Mycobacterium phage Gizmo
Mycobacterium phage Pleione
Mycobacterium phage ScottMcG
Mycobacterium phage Spud
Mycobacterium phage Bxz2
Pseudomonas phage JBD88a
Pseudomonas phage MP22
Burkholderia phage BcepMigl
Campylobacter phage CP21
Rhodococcus phage RGL3
Aeromonas phage PX29
Staphylococcus phage 2638A
Vibrio phage nt−1
Mycobacterium phage Thibault
Prochlorococcus phage P−HM2
Mycobacterium phage MoMoMixon
Cyanophage MED4−213
Choristoneura occidentalis granulovirus
Streptococcus pneumoniae bacteriophage MM1 complete genome
Enterobacteria phage RB14
Escherichia phage wV7
Lactobacillus phage AQ113
Escherichia phage vB_EcoM_PhAPEC2
Phage phi OH2 DNA
Yersinia phage L−413C
Staphylococcus phage 80alpha
Staphylococcus phage 53
Salmonella phage c341
Lactococcus prophage bIL312
Enterococcus phage phiFL1A
Enterobacteria phage P2
Enterobacteria phage fiAA91−ss
Enterobacteria phage If1
Brochothrix phage A9
Synechococcus phage S−CAM1 genomic sequence
Streptococcus prophage 315.4
Streptococcus prophage 315.2
Streptococcus prophage 315.1
Streptococcus phage phi3396
Streptococcus phage IC1
Streptococcus phage 858
Streptococcus phage 2972
Streptococcus phage 20617 complete prophage genome
Staphylococcus phage 42e
Phage cdtI DNA
Listeria phage A118
Listeria phage A006
Lactococcus phage phiL47
Lactobacillus prophage phiadh
Lactobacillus phage phiJL−1
Lactobacillus phage phig1e
Lactobacillus phage LF1
Lactobacillus phage Lb338−1
Lactobacillus phage KC5a
Enterococcus phage phiEF24C
Enterobacteria phage P1
Enterobacteria phage mEp460
Enterobacteria phage lambda
Enterobacteria phage HK630
Aeromonas phage Aeh1
Enterobacteria phage HK629
Pseudomonas phage PAJU2
Enterobacteria phage mEpX1
Enterobacteria phage HK544
Bacteriophage N15
Cronobacter phage CR5
Xanthomonas oryzae phage OP2 DNA
Salmonella phage vB_SosS_Oslo
Klebsiella phage phiKO2
Erwinia phage Ea35−70
Enterobacterial phage mEp390
Enterobacteria phage mEp237
Enterobacteria phage epsilon15
Cronobacter phage phiES15
Cronobacter phage ENT39118
Bacteriophage HK022
Aeromonas phage 31
Bacillus phage G
Shigella phage Shfl1
Salmonella phage vB_SenS−Ent3 complete genome
Enterobacteria phage P22 virus
Pectobacterium phage ZF40
Streptococcus phage TP−J34 complete genome
Streptococcus phage K13
Streptococcus phage 7201
Streptococcus phage 5093
Acinetobacter phage Presley
Staphylococcus phage SA11
Yersinia phage phiR1−RT complete genome
Enterobacteria phage HK106
Lelliottia phage phD2B
Streptococcus phage M102
Streptococcus phage DCC1738
Staphylococcus phage Twort
Sphingomonas phage PAU
Enterobacteria phage mEp235
Enterobacteria phage HK97
Enterobacteria phage HK446
Bacillus phage 0305phi8−36
Campylobacter phage CPX
Enterobacteria phage UAB_Phi78
Vibrio phage VH7D
Streptococcus phage SMP
Streptococcus phage Sfi21
Streptococcus phage Sfi19
Streptococcus phage phiBHN167 complete genome
Streptococcus phage Abc2
Enterobacteria phage HK140
Staphylococcus phage phiPVL108
Synechococcus phage S−IOM18 genomic sequence
Serratia phage Eta
Escherichia phage vB_EcoS_FFH1
Lactobacillus phage LP65
Streptococcus phage TP−778L complete genome
Streptococcus phage PH10
Streptococcus phage O1205
Streptococcus phage DT1
Streptococcus phage ALQ13.2
Salmonella phage epsilon34
Mycoplasma phage phiMFV1
Enterobacteria phage JSE
Lactobacillus phage Sha1
Vibrio phage pYD38−A genomic sequence
Stx2 converting phage II DNA
Stx2−converting phage 1717
Salmonella phage Fels−1
Salmonella enterica bacteriophage SE1
Ralstonia phage RSL1
Phage Gifsy−1
Enterobacteria phage YYZ−2008
Enterobacteria phage ST64T
Enterobacteria phage ST104 DNA
Enterobacteria phage P4
Enterobacteria phage Min27
Enterobacteria phage BP−4795
Bacteriophage RB32
Enterobacteria phage 933W
Yersinia phage PY54
Yersinia phage phiR1−37
Xanthomonas phage vB_XveM_DIBBI
Streptococcus thermophilus bacteriophage Sfi11
Streptococcus phage PH15
Salmonella phage SPN9CC
Salmonella phage SPN3UB
Salmonella phage SPN1S
Salmonella phage RE−2010
Pseudomonas phage OBP
Phage Gifsy−2
Pandoravirus salinus
Mycobacterium phage PegLeg
Escherichia phage HK75
Enterobacterial phage mEp234
Enterobacteria phage ime09
Aeromonas phage CC2
Enterobacteria phage Fels−2
Streptococcus prophage 315.6
Shigella phage SfIV
Enterobacteria phage 9g
Salmonella phage vB_SemP_Emek
Vibrio phage PWH3a−P1 genomic sequence
Streptococcus pyogenes strain NIH1 putative methionine sulfoxide reductase gene
Staphylococcus phage 69
Shigella phage SfII
Ostreococcus tauri virus 1 complete genome
Lactococcus phage P087
Lactobacillus phage phiJB
Lactobacillus phage phiAT3
Escherichia phage TL−2011b
Escherichia phage phiV10
Escherichia phage ECML−134
Enterobacteria phage SfV
Enterobacteria phage Sf6
Cyanophage NATL1A−7
Cronobacter phage vB_CsaM_GAP32
Cronobacter phage vB_CsaP_GAP52

Supplementary Figure 6. Bacteriophages in the gut microbiome of ACVD and other diseases.

Differentially enriched phages in the disease cohorts. Red, case-enriched; green, control-enriched. +, q-value < 0.05; *, q-value < 0.01, FDR controlled Wilcoxon rank-sum test.

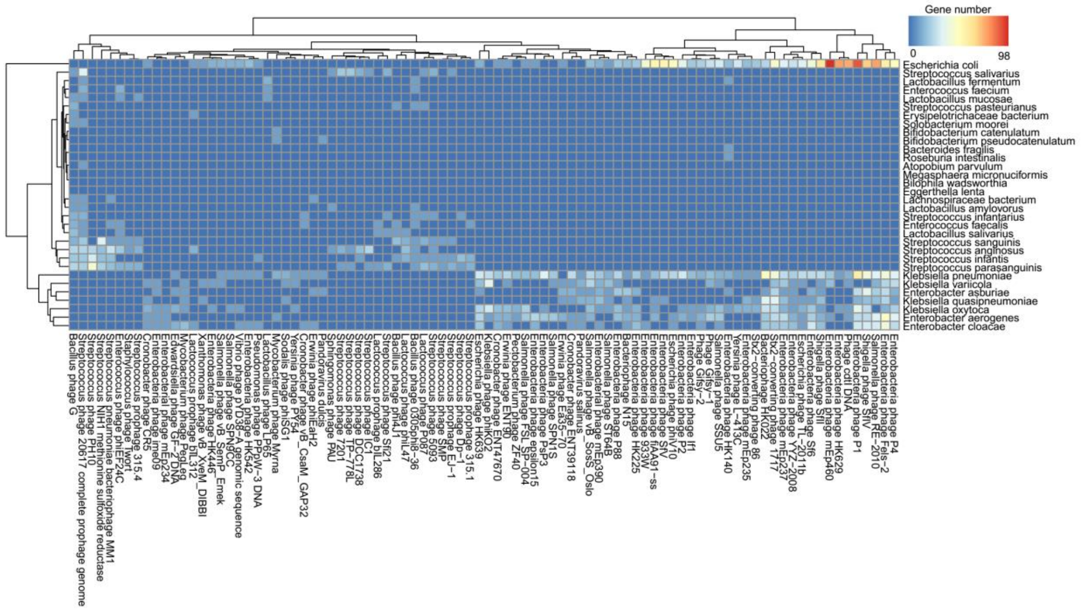

Supplementary Figure 7. Phage genes in the ACVD microbiome.

PanPhlAn was used to determine the presence or absence of genes in each sample that belong to bacterial species identified as differentially enriched MLGs (**Fig. 2**, **Supplementary Data 3**). Colored according to the number of genes that showed a significant difference in occurrence between ACVD cases (n=218) and controls (n=187). Fisher test, q-value<0.05.

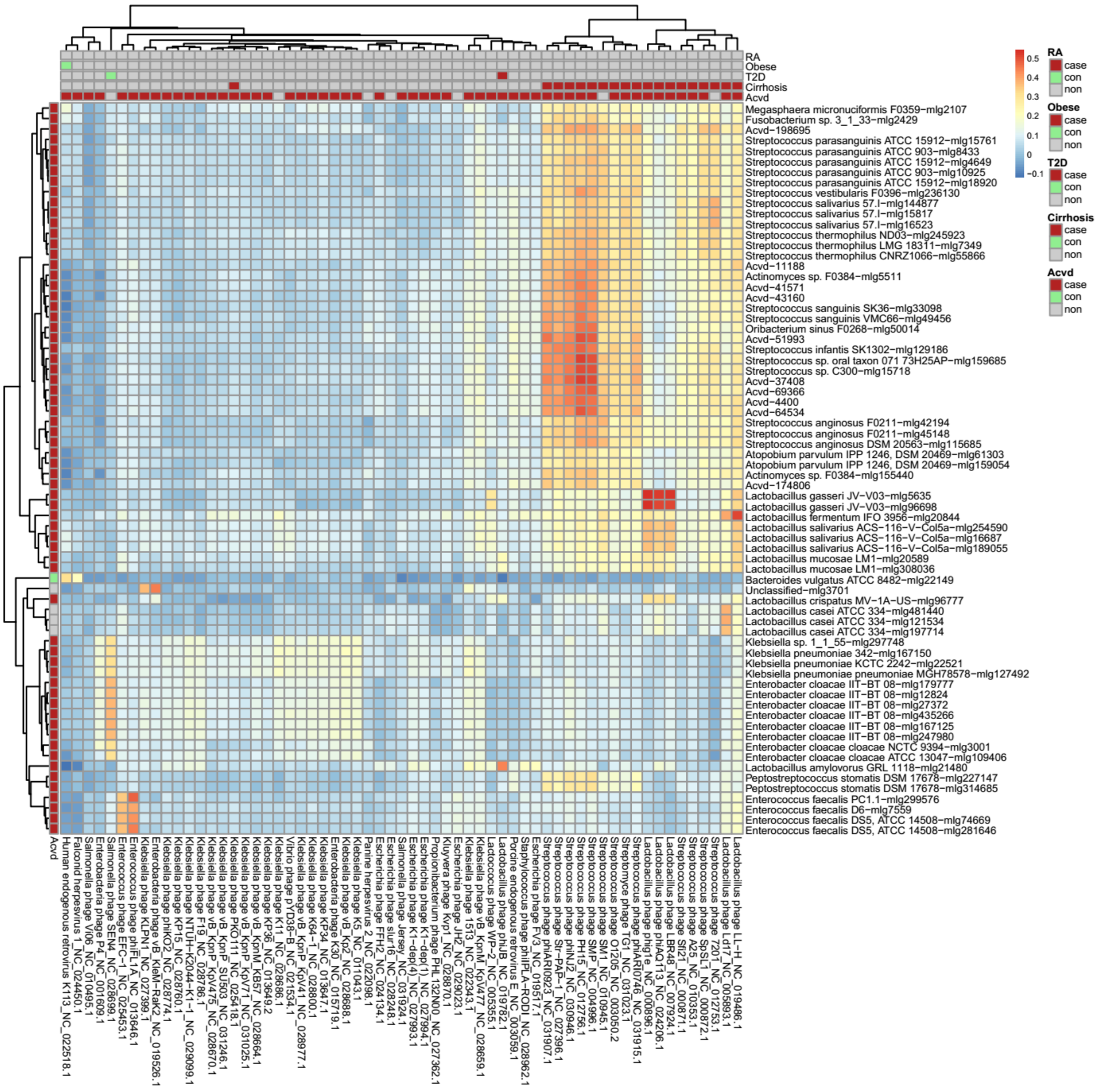

Supplementary Figure 8. Correlation between phages and bacteria in the ACVD microbiome.

Spearman's correlation was performed between the relative abundances of phages and MLGs. Blue, negative correlation; red, positive correlation. The heatmap was hierarchically clustered on both axes. Red, case-enriched; green, contro-enriched; grey, no significant difference, p-value >= 0.05, Wilcoxon rank-sum test.

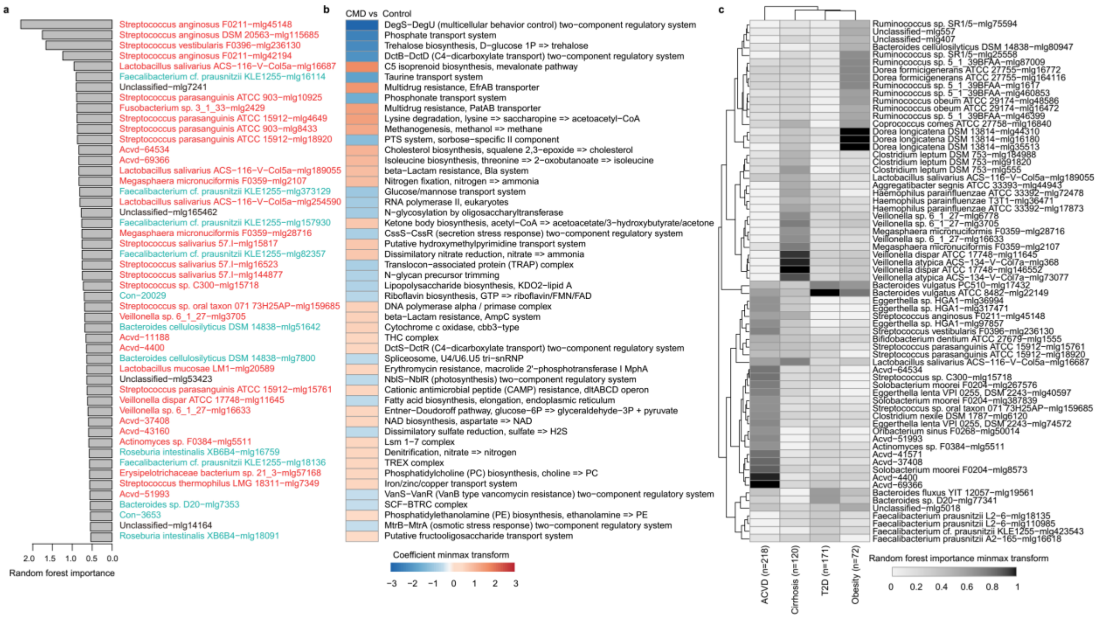

Supplementary Figure 9. Gut microbiome-based identification of Cardiometabolic diseases.

(a) MLGs important for identifying individuals with and without CMDs (n = 582 cases, 541 controls, without the RA cohort). The data were down-sampled to the same sample size for each CMD. The MLGs were ordered according to their importance for the two-way random forest classifier. X-axis represents random forest importance. Red, ACVD-enriched; cyan, control-enriched; black, no significant difference, Wilcoxon rank-sum test, q-value ≥ 0.05. (**b**) KO modules important for identifying individuals with and without CMDs. Coefficients from bootstrapping cross-validation group LASSO are plotted. Modules with larger absolute values of the coefficients appear before those with smaller absolute values of the coefficients. (**c**) MLGs important for identifying each CMD (n = 582 cases, 541 controls, without the RA cohort). Colored according to the MLGs' random forest importance after min-max transformation.

Supplementary Table 1. PERMANOVA for the influence of ACVD on gut microbial gene abundance, without (upper) and with (lower) adjustment for medication. Bray-Curtis distance, 99999 permutations.

Non adjustment						
	**Df**	**SumsOfSqs**	**MeanSqs**	**F.Model**	**R2**	**P value**
**CHD**	1	2.902	2.902	20.182	0.048	0.000
**Residuals**	403	57.944	0.144		0.952	
**Total**	404	60.845			1.000	
**Adjust drug effect permanova**						
**Drug (Drug ID_number of samples with the drug)**	**Df**	**SumsOfSqs**	**MeanSqs**	**F.Model**	**R2**	**P value**
**Clopidogrel.Hydrogen.Sulphate.Tablets_117**	1	0.836	0.836	6.277	0.017	0.000
**Aspirin_33**	1	0.209	0.209	1.573	0.004	0.137
**Atorvastatin_25**	1	0.355	0.355	2.667	0.007	0.021
**Esomeprazole_9**	1	0.050	0.050	0.379	0.001	0.926
**Isosorbide.Mononitrate_11**	1	0.167	0.167	1.257	0.003	0.246
**Perindopril_15**	1	0.150	0.150	1.128	0.003	0.311
**Bisoprolol_9**	1	0.122	0.122	0.918	0.003	0.449
**Metoprolol_19**	1	0.354	0.354	2.658	0.007	0.022
**Isosorbide.dinitrate_10**	1	0.215	0.215	1.613	0.004	0.129
**Acarbose_7**	1	0.324	0.324	2.434	0.007	0.034
**Captopril.Tablets_6**	1	0.239	0.239	1.796	0.005	0.096
**Estazolam_6**	1	0.275	0.275	2.066	0.006	0.062
**Nitroglycerin.Tablets_8**	1	0.206	0.206	1.551	0.004	0.144
**Potassium_chloride_32**	1	0.428	0.428	3.213	0.009	0.009
**Acvd**	1	1.532	1.532	11.511	0.031	0.000
**Residuals**	325	43.263	0.133		0.888	
**Total**	340	48.726			1.000	

Supplementary Table 2. Influence of drugs on the gut microbiome. PERMANOVA was performed for each drug in relation to the relative abundances of gut microbial genes in each sample. Bray-Curtis distance, 999 permutations. The categories of the drugs are: A: Drugs for acid related disorders; D: Drugs used in diabetes; H: Drugs used in high blood pressure; L: Drugs for lipid modifying; P: Drugs used in Psycholeptics; S: Supplements; T: Antithrombotics drugs; V: Vasodilators used in cardiac diseases.

Drug ID_Samples number with the drug	Df	Sums Of Sqs	MeanSqs	F.Model	R2	P value	Samples number with the drug	Drug classfication
**Clopidogrel Hydrogen Sulphate_117**	1	0.0002	0.0002	1.1966	0.0078	0.1240	117	T
**Aspirin_33**	1	0.0002	0.0002	0.9434	0.0062	0.6050	33	T
**Atorvastatin_25**	1	0.0002	0.0002	1.2903	0.0084	0.0670	25	L
**Esomeprazole_9**	1	0.0001	0.0001	0.8310	0.0054	0.8420	9	A
**Isosorbide Mononitrate_11**	1	0.0002	0.0002	1.1197	0.0073	0.2170	11	V
**Potassium Citrate_14**	1	0.0002	0.0002	1.0647	0.0070	0.3110	14	S
**Perindopril_15**	1	0.0002	0.0002	1.1407	0.0074	0.1600	15	H
**Heparin Sodium_10**	1	0.0001	0.0001	0.8936	0.0058	0.7250	10	T
**Bisoprolol_9**	1	0.0001	0.0001	0.8893	0.0058	0.7460	9	H
**Fondaparinux**	1	0.0002	0.0002	1.4631	0.0095	0.0170	10	T

**Sodium_10**								
**Metoprolol_19**	1	0.0002	0.0002	1.3699	0.0089	0.0400	19	H
**Insulin aspart_6**	1	0.0001	0.0001	0.8790	0.0057	0.7640	6	D
**Isosorbide dinitrate_10**	1	0.0002	0.0002	1.1369	0.0074	0.1750	10	V
**Acarbose_7**	1	0.0002	0.0002	1.3913	0.0091	0.0330	7	D
**Captopril_6**	1	0.0002	0.0002	0.9346	0.0061	0.5940	6	H
**Estazolam_6**	1	0.0002	0.0002	1.0186	0.0067	0.3980	6	P
**Nitroglycerin_8**	1	0.0002	0.0002	1.1029	0.0072	0.2570	8	V
**Potassium Chloride_32**	1	0.0002	0.0002	1.1942	0.0078	0.1290	32	S

Supplementary Table 3. Distinguishing CMDs using MLGs. Prediction error according to the RFCV model in **Fig. 3a**.

	**Predict Control**	**Predict Acvd**	**Predict Cirrhosis**	**Predict T2D**	**Predict Obesity**	**Error**
**True Control**	352	62	25	74	38	0.361
**True Acvd**	34	156	12	11	5	0.284
**True Cirrhosis**	17	2	98	3	0	0.183
**True T2D**	38	9	7	114	3	0.333
**True Obesity**	17	10	1	4	40	0.444
