# Supplementary material for: The gut microbiome in atherosclerotic cardiovascular disease
Source: Nat Commun. 2017 Oct 10;8:845. doi: 10.1038/s41467-017-00900-1 (PMC5635030; doi:10.1038/s41467-017-00900-1)
Supplement: Supplementary file 2 — Description of Additional Supplementary Files [file 41467_2017_900_MOESM2_ESM.pdf]

Description of Additional Supplementary Files

File Name: Supplementary Data 1
Description: Phenotypes of the ACVD cohort.

File Name: Supplementary Data 2
Description: Statistics for the metagenomic shotgun sequencing data of the ACVD cohort.

File Name: Supplementary Data 3
Description: MLGs of the ACVD cohort. Clusters containing > 100 genes were annotated according to available bacteria and archaea genomes, as described previously (Qin et al. 2012).

File Name: Supplementary Data 4
Description: AUC and Youden's index of random forest classifiers for ACVD samples stratified by two drug combinations. Performed as in Fig. 5b, on patients with and without either drugs, instead of single drugs.

File Name: Supplementary Data 5
Description: Abundance differences of individual KOs in the disease cohorts. P-values from Wilcoxon rank-sum test, q-value to control for multiple testing.

File Name: Supplementary Data 6
Description: Reporter score for KO modules in the cohorts. rs >0, ACVD-enriched; rs < 0, control-enriched.

File Name: Supplementary Data 7
Description: Reporter score for KO pathways in the cohorts. rs >0, ACVDe-enriched; rs < 0, control-enriched.

File Name: Supplementary Data 8
Description: Disease probability according to the RFCV model in Fig. 3.
